# Is the Colonisation of *Staphylococcus aureus* in Pets Associated with Their Close Contact with Owners?

**DOI:** 10.1371/journal.pone.0156052

**Published:** 2016-05-26

**Authors:** Karolina Bierowiec, Katarzyna Płoneczka-Janeczko, Krzysztof Rypuła

**Affiliations:** Division of Infectious Diseases and Veterinary Administration, Department of Epizootiology with Clinic of Birds and Exotic Animals, Faculty of Veterinary Medicine, Wrocław University of Environmental and Life Sciences, Wrocław, Poland; Rockefeller University, UNITED STATES

## Abstract

In human beings and animals, staphylococci constitute part of the normal microbial population. *Staphylococcus aureus* could be classified as an opportunistic pathogen because the bacteria are noted in clinically healthy individuals, but when the immune system becomes compromised, they can also cause a wide range of infections. The objective of this study was to test the hypothesis that cats who are in close contact with their owners are at the greatest risk of being colonised with *S*. *aureus*. Two groups of cats were investigated: single, pet (domestic) cats that do not have outdoor access; and a local population of feral cats living in urban areas. The prevalence of *S*. *aureus* in domestic cats was 19.17%, while it’s prevalence in the feral cat population was only 8.3%; which was statistically significant. Analysis of antibiotic resistance, at the genotypic as well as phenotypic level, showed that *S*. *aureus* isolates from pet cats were more likely to harbour antibiotic resistant determinants. The prevalence of methicillin-resistant *Staphylococcus aureus* (MRSA) in households was 10.21%, while in feral cats it was only 1.4%. In conclusion, this study has revealed a correlation between close contact with humans and a higher risk of the cats being colonised with *S*. *aureus* and harbouring the antibiotic resistant determinants.

## Introduction

In humans and animals, staphylococci constitute part of the normal microbial flora. There are several different definitions of what constitutes an animal’s normal bacterial flora. There are the”symbionts”, which benefit themselves and the host; “commensals”, which do not benefit the host but are harmless; and the “opportunists”–typically non-pathogenic microorganisms that act as a pathogen in certain circumstances [1]. According to the aforementioned definitions, *Staphylococcus aureus* could be classified as an opportunistic pathogen for both humans and animals. Although the bacteria are found in clinically healthy individuals, they can cause a wide range of infections when the immune system becomes compromised or select comorbidities are associated (congestive heart failure, diabetes, pulmonary disease and renal failure) [2, 3].

The composition of normal flora on an organism depends on the species, feed and environment, including population density. Nevertheless, *S*. *aureus* is the most frequently isolated coagulase positive *Staphylococcus* (CPS) from the anterior nares and temporarily from the skin of humans, whereas coagulase negative staphylococci (CNS), mainly *Staphylococcus epidermidis*, are dominant on the skin [4]. Similarly, for cats CNS are the major species in this tissue and mucosa’s natural flora. The most frequently observed is *S*. *felis*, and seldom CPS such as *S*. *pseudointermedius*, but in some environments *S*. *aureus* is also observed [5].

Investigation of the nasal carriage is used in epidemiology as a marker of *S*. *aureus* exposure with an increased risk of infection in humans [6] *inter alia*, of some skin diseases [7], wound colonisation [8], surgical site infections [9] or respiratory tract infections [10].

In animals, colonisation of the nares with *S*. *aureus* is usually used to assess human exposure to livestock or pet- associated *S*. *aureus*, mainly methicillin- resistant *S*. *aureus* strains (MRSA). The circulation of *S*. *aureus* clones vary between hosts, environments and countries. Some lineages are described in which they are well adapted to their respective host whereas others seem to have a broader host range. For example, the majority of isolates from nares in swine belong to lineages CC30 and CC398 [11], but only ST433is host-specific [12]. Originally CC398 was of human origin and now it is adapted to livestock such as pigs, poultry and cattle or other animals: horses and pets. A similar situation was observed in poultry where CC5 was adapted from humans. [13, 14]. In ruminant mastitis isolates are frequently associated with CC8, CC97, CC126, CC130, CC133 and CC705; whereas rabbit infections mostly belong to ST21 [15]. Clonal complexes CC5, CC8, CC22, CC30 and CC45 are more frequently associated with hospital-acquired infections [16]. Two MRSA clones: ST22 and ST239, have dominated globally in hospital settings [17]; while in the community at large mainly ST8 in the United States and ST80 and ST22 in Europe have been found [18]. There is currently no evidence for the pet-adapted *S*. *aureus*. Companion animals usually share their environment with humans, which could indicate a reduced opportunity for host adaptation [19]. MRSA lineages isolated from infected companion animals often mirror typical human epidemic strains circulating in the same region: ST22 in the United Kingdom, Germany, Portugal and ST59 in China [20, 21]. In cats and dogs in Germany, the predominant lineages were CC22 and CC5 followed by CC398 and CC8 [22].

The aim of the study was to test the hypothesis that cats that are in close contact with their owners are at the greatest risk of increased colonisation with *S*. *aureus*. To assess this assumption, a cohort study was designed, which studied two groups of cats: the first was the pet cat group primarily in contact only with their owners; the second group was comprised of free-living, wild domestic cats living in the city. This study also provides detailed information on *S*. *aureus* strains isolated from both groups and on the drug resistance profile of these bacteria to different antibiotic classes using both phenotypic and molecular methods.

## Materials and Methods

### Study population and sampling procedures

There were two groups of cats examined. The first group, comprised of pet cats, were recruited as part of a randomised control trial that targeted clinically healthy cats sourced from the city of Wrocław area. This group’s primary inclusion criterion was the cat owner’s statement that the pet was kept in Wrocław without outdoor access and that the cat was the only animal present in the household. The second group were free-living cats within the city of Wrocław. These animals did not have contact with humans and were sampled during a trap, neuter and release (TNR) programme for the humane control of the feral cat population by the Department of Reproduction and Clinic of Farm Animals, Faculty of Veterinary Medicine in Wrocław. All the animals that qualified for the surgery in this programme were clinically healthy. The health status of each animal from both groups was assessed by way a diagnostic examination in conjunction with a clinical examination.

Four swabs were taken from each cat in both groups, as follows: from the conjunctival sacs, nares, anus and skin (groin). The material was collected by a veterinary physician and placed into 2 ml of liquid brain-heart infusion bullion (BHI) (Graso Biotech, Poland).

The research outline was submitted to the II Local Ethics Committee for Animal Experiments in Wrocław. Due to the non-invasive procedure of the samples collection, the Ethics Committee qualified the study as research that did not require ethics committee approval.

### Sample identification

The tubes with swabs in 2 ml BHI were incubated at 37°C for 24 hours, then one microlitre of BHI was subcultured onto a mannitol salt broth and blood agar (Graso Biotech, Poland). The plates were incubated at 37°C for the next 24 hours. The preliminary identification of isolates was performed according to colony morphology, gram staining and the detection of enzyme production (coagulase tube test; IBSS Biomed, Poland). All the suspected colonies were further identified using molecular methods.

The purification of DNA was conducted using the manual method of phenol/chloroform employing an initial digestion by lysozyme (Sigma-Aldrich, USA) as described previously by Bania et al. [23]. Isolates were confirmed by a polymerase chain reaction using *S*. *aureus nuc* gene specific primers, which encode thermonuclease [24]. In addition, 25% of isolates were confirmed as *S*. *aureus* using BLAST analysis from the 16S RNA PCR product (http://rdna4.ridom.de). The obtained sequences were identified by comparison with sequences available in the GenBank database, using a BLAST search algorithim (http://blast.ncbi.nlm.nih.gov/Blast.cgi).

Isolates were identified as MRSA by detection of the *mec*A or *mec*C gene using PCR [25, 26]. Amplification of the short sequence repeat region of the *spa* gene was conducted using specific primers [27]. The thermal cycling conditions were set up according to Shopsin et al. [28] and the completed reaction mixtures were sequenced by Macrogen (Netherlands). The sequences were analysed using the Ridom SpaServer (http://spa.ridom.de).

### Antibiotic resistance

All the isolates of *S*. *aureus* were screened for antibiotic susceptibility using the disc diffusion method and MIC with the E-test. The antimicrobial disc diffusion tests were as follows (μg/disc): penicillin G (10), cefoxitin (30), erythromycin (15), clindamycin (2), gentamicin (10), tetracycline (30), norfloxacin (10), chloramphenicol (30), mupirocin (200), fusidic acid (10), vancomycin (30), tigecycline (15) and linezolid (30) (Mast Diagnostics, UK). The double-disc diffusion test (D-test) was performed on all isolates to detect inducible clindamycin resistance. The interpretation of the test was as follows: a flattening of the inhibition zone around the clindamycin disc near the erythromycin disc indicated that erythromycin had induced clindamycin resistance (iMLS_B_). Erythromycin and clindamycin resistance characterised the phenotype cMLSB. The phenotype (MS_B_) was characterised by clindamycin susceptibility and erythromycin resistance, with a negative D-test [29].

The MIC tests (MIC Test Strip, Liofilchem, Italy) were as follows: oxacillin (0.16–256), penicillin (0.016–256), erythromycin (0.016–256), clindamycin (0.016–256), genatmicin (0.064–1024), tetracycline (0.016–256), mupirocin (0.064–1024), fusidic acid (0.016256) vancomycin, tigecycline (0.016–265) and linezolid (0.016–256). Antimicrobial-resistant phenotyping of isolates was performed and interpreted according to the Clinical and Laboratory Standards Institute document M100-S24 [30]. Evidence of tigecycline, mupirocin and fusidic acid were interpreted according to the protocol used in recent studies [31–33].

Antimicrobial-resistant genotypes of isolates were identified using PCR. The presence of genes involved in resistance to penicillinase (*bla*Z), aminoglycosides (*aac(6’)Ie-aph(2”)Ia*), ß-lactamase (*mec*A, *mec*C), glycopeptides (*van*A and *van*B), macrolide-lincosamide-streptogramins (*erm*A, *erm*B and *erm*C), tetracyclines (*tet*K, *tet*L, *tet*M and *tet*O), mupirocine (*mup*A) and fusidic acid (*fus*B, *fus*C, *fus*D) was determined using PCR amplification [25, 26, 34–37]. The reaction volume was 25 μL, containing 0.2 μL of each primer, 2.5 μL of DreamTaq green buffer, 1 U of DreamTaq DNA polymerase (Thermo Scientific, Lithuania) and 1 μL of matrix DNA. Electrophoresis was performed on 2% agarose gel with Midori Green (Nippon Genetics Europe, Germany). Selected PCR products were sequenced (Macrogen Netherlands) and analysed using the BLAST method.

### Statistical methods

To calculate the prevalence and confidence intervals in both groups of cats, the bootstrap method was used. This was performed by drawing 78 replacement cats (each of which had an equal probability) from a pool of 78 animals in the pet cat group, and 72 cats (with equal probability each) from a pool of 72 animals in the group of free-living domestic cats. This process was performed with 10 000 reiterations.

The characteristics of the cats were compared to scores of *S*. *aureus* colonisation and antibiotic resistance. Data were entered into a computerised database and analysed using the Shapiro–Wilk test, the Wilcoxon test, the Kruskal–Wallis test, 2 × 2 contingency tables and bootstrapped chi-square tests. *P* <0.05 was considered indicative of a statistically significant association. Residue tables were constructed for statistically significant results and used to detect existing relationships between characteristics. A residue table shows the frequency distribution of the values of the dependent variable, given the occurrence of the values of the independent variable. Statistical analyses were carried out using the R Statistical Package (*v*. 2.11.1).

## Results

**Fig 1:**
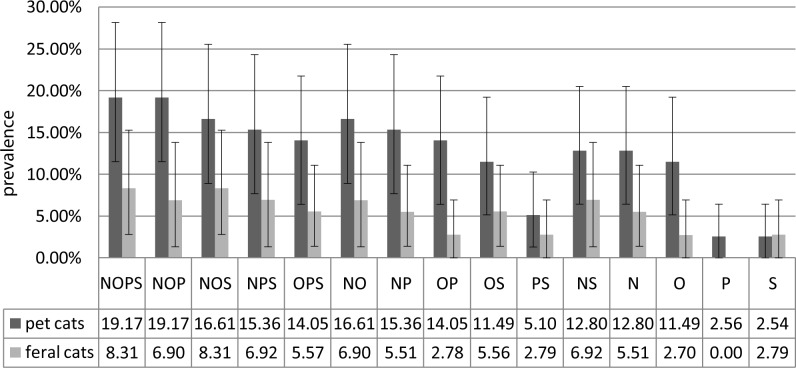
Prevalence of *S*. *aureus* in pet cats and feral cats, including combinations of sampling places. Sampling places: nares (N); conjunctival sacs (O); anus (P); skin (S). For each combination of sampling places a confidence interval was marked which was calculated using the bootstrap method.

**Fig 2:**
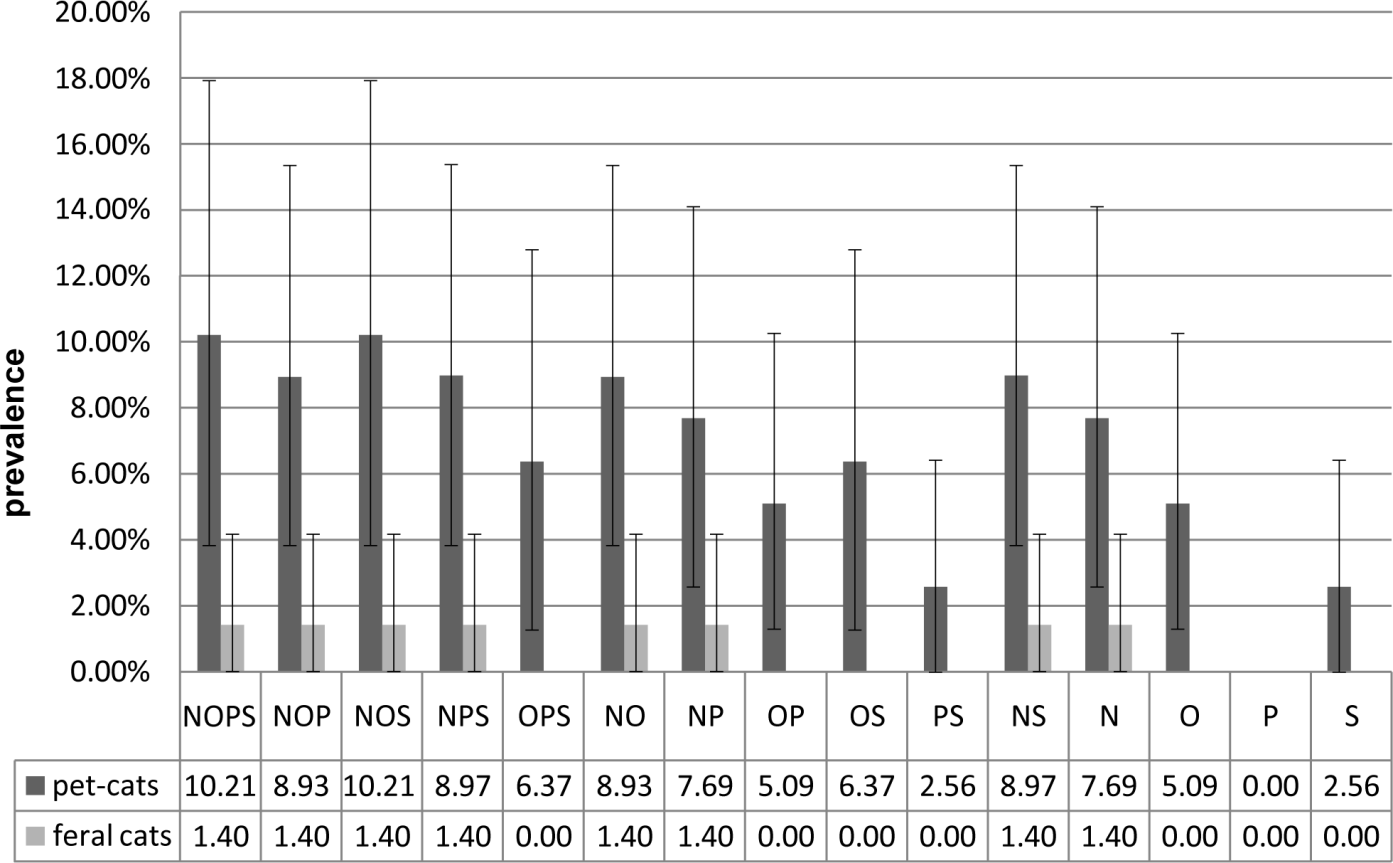
Prevalence of MRSA in pet cats and feral cats, including combinations of sampling places. Sampling places: nares (N); conjunctival sacs (O); anus (P); skin (S). For each combination of sampling places a confidence interval was marked which was calculated using the bootstrap method.

A total of 150 cats were examined from January 2013 to November 2014 at the Department of Epizootiology and Clinic of Bird and Exotic Animals, Faculty Veterinary Medicine, Wrocław University of Environmental and Life Sciences, Poland. Cats were assigned to two groups: the pet cats without outdoor access (n = 78; 51.3% female and 48.7% male) and feral cats living in the urban area (n = 72; 69.4% female and 30.6% male). The animals were considered positive if *S*. *aureus* was isolated from any site (skin, conjunctival sacs, anus or nares). The research included an examination of 32 S. *aureu*s strains from both groups (24 and 8 from pet cats and feral cats respectively). The average prevalence of *S*. *aureus* among pet cats and feral cats was determined using the bootstrap method was 19.17% (11.5–28.21%) and 8.3% (2.78–15.28%), respectively. The difference between groups was statistically significant (*P* = 0.044). *S*. *aureus* isolates were identified as a MRSA when the *mec*A gene was present, regardless of whether the oxacillin MICs were <4 μg/L. The presence of the *mec*C gene was not identified in any of the investigated isolates. The research included an examination of 11 MRSA strains from both groups (10 and 1 from pet cats and feral cats respectively). The prevalence of MRSA isolates was 10.21% (3.85–17.95%) for pet cats and 1.4% (0–4.17%) for feral cats and the difference between the two groups was statistically significant (*P* = 0.0112). There was more than one *S*. *aureus* isolate identified in 35% of all cats colonised with *S*. *aureus*. In all of these cases, *S*. *aureus* were isolated from the nares and usually from the conjunctival sacs. The nares turned out to be the most sensitive anatomical location to detect *S*. *aureus* colonisation in both groups, as well as for total *S*. *aureus* isolates and for MRSA. The combinations of prevalence using different sampling places and a comparison between groups are presented in Figs 1 and 2.

Any genetic determinants of resistance to the following were not detected: vancomicin (*van*A and *van*B genes), mupirocin (*mup*A gene) and fusidic acid (*fus*B, *fus*C and *fus*D genes) in both groups. Comparisons of residual results from PCR are presented in Table 1. The results of phenotypic resistance to antibiotics using an E-test are shown in Table 2. There were no phenotypic resistance isolates to mupirocin, linezolid, tigecycline, fusidic acid, vancomycin, norfloxacin and chloramphenicol identified. Only inducible resistance to clindamycin (iMLS_B_) was observed in one isolate from each group (the pet cat group and feral cat group).

**Table 1:** Percentage of genetic determinants of the antibiotic resistance among isolates of *S*. *aureus*.

t	Genetic determinants of the antibiotic resistance									
category	*bla*Z	*mec*A	*aph*	*erm*A	*erm*B	*erm*C	*tet*K	*tet*L	*tet*M	*tet*O
	*p = 0*.*2466*	*0*.*2116*	*p<0*.*001*	*p<0*.*001*	*p<0*.*001*	*p = 0*.*5507*	*-*	*p = 0*.*5465*	*p<0*.*001*	*p<0*.*001*
***pet cats***	100%	41.67%	91.67%	91.67%	100%	16.67%	100%	16.67%	91.67%	75%
***feral cats***	87.5%	12.5%	-	12.5%	-	-	100%	-	-	-

p–probability value

**Table 2:** Percentage of antibiotic resistance according MIC test in all isolated *S*. *aureus* strains.

category	peinicilin	oxacilin	gentamycin	erythromycin	tetracycline
pet cats	58.33%	8.33%	4.17%	4.17%	4.17%
feral cats	37.5%	-	-	12.5%	-

Isolates were characterised into 14 and 6 *spa* types in the pet cats group and the feral cats respectively. The most frequently observed were t008 (20.83%) in pet cats, and t755 (25%) and t005 (25%) in feral cats. No correlations were observed between *spa* types and the anatomical location of *S*. *aureus* isolation or the affiliation of the cat to any of the investigated groups. The comparison of *spa* types isolated in both groups is presented in Table 3.

**Table 3:** Diversity of *spa* types and antibiotic resistance patterns in pet cat and feral cat groups.

spa type / MLST*	ISOLATE	GENOTYPIC RESISTANCE / PHENOTYPIC RESISTANCE
**t002** /ST5; ST231	PC/N	*bla*Z, *mec*A, *erm*A, *erm*B, *tet*(K), *tet(*M) / PN
	PC/O	*bla*Z, *mec*A, *aac(6’)Ie-aac(6’)Ie-aph(2”)Ia(2”)Ia*, *erm*A, *erm*B, *tet*(K) / PN
**t005** /ST22;ST23; ST60/	PC/N	*bla*Z, *aac(6’)Ie-aph(2”)Ia*, *erm*A, *erm*B, *tet*(K), *tet*(M) / PN
	PC/O	*bla*Z, *mec*A, *aac(6’)Ie-aph(2”)Ia*, *erm*A, *erm*B, *erm*C, *tet*(K), *tet*(L), *tet*(M), *tet*(O) / PN
	FC/N	*bla*Z, *tet*(K)
	FC/S	*bla*Z, *erm*A, *tet*(K) / PN
**t008** /ST8; ST247; ST250; ST254/	PC/N	*bla*Z, *aac(6’)Ie-aph(2”)Ia*, *erm*A, *erm*B, *tet*(K), *tet*(M) / PN
	PC/O	*bla*Z, *aac(6’)Ie-aph(2”)Ia*, *erm*A, *erm*B, *tet*(K), *tet*(M) / PN
	PC/N	*bla*Z, *aac(6’)Ie-aph(2”)Ia*, *erm*A, *erm*B, *erm*C, *tet*(K), *tet*(M), *tet*(O) / PN
	PC/P	*bla*Z, *aac(6’)Ie-aph(2”)Ia*, *erm*A, *erm*B, *tet*(K), *tet*(M), *tet*(O) / PN
	PC/P	*bla*Z, *aac(6’)Ie-aph(2”)Ia*, *erm*A, *erm*B, *tet*(K), *tet*(M), *tet*(O) / PN
	FC/S	*bla*Z, *tet*(K) / PN
**t037** /ST239; ST240; ST241/	PC/N	*bla*Z, *mec*A, *erm*A, *erm*B, *tet*(K), *tet*(M), *tet*(O) / PN, FOX, GN, ER, TET
**t091** /ST7; ST943; ST2632/	PC/O	*bla*Z, *erm*A, *erm*B, *tet*(K), *tet*(0)
**t189** /ST188; ST2635/	PC/N	*bla*Z, *mec*A, *aac(6’)Ie-aph(2”)Ia*, *erm*A, *erm*B, *tet*(K), *tet*(M), *tet*(O)
	PC/N	*bla*Z, *aac(6’)Ie-aph(2”)Ia*, *erm*A, *erm*B, *tet*(K), *tet*(L), *tet*(M), *tet*(O)
**t304** /ST6/	FC/N	*bla*Z, *mec*A, *tet*(K) / PN
**t521**	PC/N	*bla*Z, *mec*A, *aac(6’)Ie-aph(2”)Ia*, *erm*A, *erm*B, *tet*(K), *tet*(M), *tet*(O)
**t700**	PC/O	*bla*Z, *aac(6’)Ie-aph(2”)Ia*, *erm*A, *erm*B, *erm*C, *tet*(K), *tet*(M), *tet*(O) / PN
**t775**	FC/N	*tet*(K)
	FC/N	*bla*Z, *tet*(K)
	PC/O	*bla*Z, *mec*A, *aac(6’)Ie-aph(2”)Ia*, *erm*A, *erm*B, *erm*C, *tet*(K), *tet*(M), *tet*(O)
**t852** /ST22/	PC/O	*bla*Z, *aac(6’)Ie-aph(2”)Ia*, *erm*B, *tet*(K), *tet*(M), *tet*(O)
**t8420**	PC/N	*bla*Z, *aac(6’)Ie-aph(2”)Ia*, *erm*A, *erm*B, *tet*(K), *tet*(M)
	PC/S	*bla*Z, *aac(6’)Ie-aph(2”)Ia*, *erm*B, *tet*(K), *tet*(L), *tet*(M), *tet*(O)
	FC/O	*bla*Z; *tet*(K) / GN
**t6989**	FC/O	*bla*Z, *tet*(K)
**t7482**	PC/O	*bla*Z, *mec*A, *aac(6’)Ie-aph(2”)Ia*, *erm*A, *erm*B, *tet*(K), *tet*(M), *tet*(O)
**t11024**	PC/S	*bla*Z, *mec*A, *aac(6’)Ie-aph(2”)Ia*, *erm*A, *erm*B, *tet*(K), *tet*(M), *tet*(O) / PN
**t11455**	PC/O	*bla*Z, *aac(6’)Ie-aph(2”)Ia*, *erm*A, *erm*B, *tet*(K), *tet*(M), *tet*(O) / PN
	PC/N	*bla*Z, *aac(6’)Ie-aph(2”)Ia*, *erm*A, *erm*B, *tet*(K), *tet*(M), *tet*(O) / PN
**t12411**	PC/N	*bla*Z, *mec*A, *aac(6’)Ie-aph(2”)Ia*, *erm*A, *erm*B, *erm*C, *tet*(K), *tet*(L), *tet*(M), *tet*(O)

There were two groups of investigated cats: feral cats (FC) and pet cats (PC). Sampling places: nares (N); conjunctival sacs (O); anus (P); skin (S). PN—penicilin, FOX—cefoxitin, GN—gentamivin, ER—erythromycin, TET—tetracycline.

* Predicted MLST STs for the *spa* types according to Ridom SpaServer—Spa-MLST Mapping (http://spa.ridom.de/mlst.shtml) or from relevant literature.

## Discussion

The possibility of transmitting zoonotic agents between humans and animals has been widely described in scientific literature. For many pathogens, pet animals play a role as a source of zoonotic infections [38]. Our study was designed to confirm that close contact with humans might cause a higher chance of colonisation with *S*. *aureus* in pets. From the comparison of the two study groups of cats–exposed and not exposed to contact with humans–we have shown a statistically significant higher prevalence of *S*. *aureus* colonisation in cats that have close contact with their human owners.

The natural bacterial flora of skin and mucosa in cats was comprised mainly of CNS such as *S*. *felis* and *S*. *simulans* [39, 40] From the CPS species, the most dominant are *S*. *pseudointermedius* and *S*. *aureus*, but the prevalence of these bacteria does vary from study to study. In a study by Hariharan et al. [39], in India, no *S*. *aureus* or *S*. *intermedius/pseuintermedius* could be found in swabs from feral cats. However, in an English study on feral and pet cats, the prevalence of CPS was comparable in both groups at about 5% [41]. In Iverson et al.’s study [42], the *S*. *aureus* prevalence was 17% but there are also reports that have shown a much higher prevalence in healthy cats of around 34–39.6% [43, 44]. Most commonly the research has studied the prevalence of *S*. *aureus* in domestic cats and little is known about its prevalence in feral cats. In this study, the prevalence of *S*. *aureus* in feral cats was found to be 8.3% and was much lower than that observed in pet cats (19.17%).

Large differences in the colonisation of cats with MRSA strains were also observed. The main factor described in scientific literature that has an influences on the prevalence value was permanent contact with the environment where the colonisation of MRSA from humans was common [45]. The prevalence in pets, which lived with human colonised with MRSA, reported by Moriss et al. was 11.6% [46]. In our study, the MRSA prevalence in pet cats was 10.21%, considerably higher compared to other findings from scientific literature where the MRSA rate was typically lower than 4% [47–49]. The households’ residents were not investigated in this study and so it is not known if the owners had an influence on the MRSA colonisation rate in these cats, or if other factors contributed to such a high MRSA prevalence.

Little is known about prevalence of *S*. *aureus* in the community of Poland, but in the hospital environment the proportion of MRSA isolates was 22.7% (ranging from 3.7 to 63.1% in individual hospitals) [50]. Relatively high MRSA prevalence may indicate a wide spread of the pathogens also in the community. We found some similarities by comparing previously described *spa* and MLST types of *S*. *aureus* isolated in Polish hospitals and *spa* types obtained in this study. *Spa* types such as: t002, t005, t008, t037, t091 and t755 were previously reported in cases of human infections in Poland [51, 52]. Additionally t008, t037, t091 and t189 were detected in a study which dealt with the epidemiology of community-associated *Staphylococcus aureus* (CA-SA) in Europe [53]. Considering the previous obtained data we can suspect that some *S*. *aureus* strains in cats were human in origin, however, further studies are required to better understand this issue.

In previous studies, other such confounding factors of high MRSA prevalence in pets were identified, including: the number of employees working at the veterinary setting where pets was being treated; antibiotic treatment prior to sampling; surgical site of infection; and surgical implants [21, 54]. Some of the pet cats under investigation, according to information obtained from the pet owners, had been previously treated (mainly by an administration of antibiotics); nevertheless, this data was not sufficient to confirm if the previously described confounding factors influencing the high MRSA prevalence.

The prevalence in the feral cats group was significantly lower at 1.4% (one isolate from the nares) in this study. Similarly, the antibiotic resistance in free-living animals was usually reported in other studies as sporadic [39, 55]. The explanation for this fact could be a low probability that chemotherapeutic treatment was conducted in such a population. Furthermore, there are only a few reports about antibiotic resistance in staphylococci isolates from feral cats and these results are divergent. Patel et al. [41] observed there was a higher antibiotic resistance in feral cats compared with pet cats, whereas in Hariharan et al.’s study [39] the antibiotic resistance was minimal. With regard to other feline infectious agents, the feral cats assessed appear to be of no greater risk to human beings or other cats than pet cats [56]. Moreover, our results show that the pet cats group is more likely to be a reservoir of *S*. *aureus* with genetic determinants of antibiotic resistance. There were only a few antibiotic resistance genes observed to be harboured by feral cats in this study. All of them are frequently present in staphylococci genomes of a different origin [57]. This is why there is difficulty in determining the origin of those genes in *S*. *aureus* that colonised the feral cats under investigation.

In the case of pet cats, there is a high probability that a larger number of determinates of antimicrobial resistance in *S*. *aureus* isolates could be connected with a previous treatment of pets and/or owners, or even previous prophylactic visits to a veterinary clinic [58–60]. Antibiotic resistant genes to commonly used chemotherapeutics were found in both human and animals isolates, such as: penicillinase, aminoglycosides, ß-lactamase, macrolides and tetracyclines, and it was only to these antibiotics that phenotypic resistance was observed. In the feral cat group, phenotypic resistance was observed to penicillin and erythromycin. Most *S*. *aureus* strains under investigation harboured genetic determinants of resistance, without showing resistance to investigated chemotherapeutics using the MIC test. The inconsistency between these results of phenotype and genotype drug susceptibility tests could be explained as differences in resistance gene expression between isolates. In none of the isolates was a phenotypic resistance observed without the genetic determinants, that would suggest the occurrence of other mechanisms of resistance [61].

The percentage of isolates which showed phenotypic resistance was comparable with similar studies conducted on healthy pets [62]; showing that a high percentage of penicillin resistant isolates is particularly common [63]. The pet cat group in this study was found to be more likely colonised with antibiotic resistant bacterial strains than feral cats. Patel et al. [41] described a reverse situation, for which the authors suggested the possibility of acquired resistance in isolates from feral cats’ resident flora via contact with environmental sources of antibiotics, such as medical waste, domestic waste (meat products) or polluted water. This could also be a cause of the appearance of resistant isolates in the healthy feral cats examined in this study and moreover the source of *S*. *aureus* strains. To the best of our knowledge, the feral cats had not previously been handled, nor had they received veterinary treatment, which could be the main source of resistance in pet cats. They could also be colonised with resistant isolates or isolates containing genetic determinates of resistance through contact with humans. The feral cats investigated in the study were free living cats residing within the city area, more often than not in cellars, warehouses and bowers. Therefore it is not possible to exclude indirect contact with humans who may have touched the same surfaces. The colonizing with *S*. *aureus* strains could also have its origin in livestock by the consumption of raw meat or contaminated pet food. For instance *spa* type t091, t015, t008 were frequently isolated in pork and poultry meat in Poland [11, 14] and in our study from cats.

There was a widely described evidence of horizontal transfer of *S*. *aureus* isolates between humans, animals and the environment habitat [64–67]. Thus, with the possibility of interspecies and environmental transfer of the pathogen, particularly pets could become a reservoir of *S*. *aureus* for humans. This is especially important in situations when animal owners suffer from remittent *S*. *aureus* infections. An ineffective treatment could result from a constant presence of the bacteria in the environment [68, 69]. Therefore, each case of MRSA or multidrug-resistant *S*. *aureus* in a human or animal patient could be a clue for doctors or veterinarians to extend the diagnostic interview and eventually perform laboratory research on the residual household residents. Moreover, in households affected by MRSA, there is a need to apply methods that counteract the spread of the bacteria. There are some available protocols used in human medicine [70, 71], but there is still a lack of similar procedures for pets. Regardless, with adherence to some of these rules, the spread and development of antibiotic resistance among *S*. *aureus* could be limited. It is not possible (or permitted) to use chemotherapeutics specified for human usage for the decolonisation of *S*. *aureus* in pets even in some countries the use of fusidic acid or mupirocin in pets is approved for the treatment of staphylococcal skin infections [72, 73]. There is a report of the successful topical decolonisation of MRSA-positive cat with ciprofloxacin and rifampin [74] which might be recommended for use in veterinary medicine. The standard protocol in counteracting the spread of the pathogen should be an adherence to personal hygiene (especially hand hygiene after handling animals) and frequent disinfection of surfaces. Pets can often clear up colonisation without drug treatment but in some cases, similar to humans, decolonisation could be transient. Therefore long-term hygiene measures in households should be applied [65, 75]. After all, pet cats could be a source of many pathogens for their owners nevertheless there are some benefits such as the presence of pets has been associated with reduction of stress and blood pressure what is associated with reduction of the risk of cardiovascular diseases [76]. Therefore pets as family members should receive the same attention as concerns the sanitising of MRSA colonisation and treatment of infections as humans.

This study is the first to confirm by using empiric research, that close human contact has an influence on the prevalence of *S*. *aureus* in cats. It can be assumed that the original direction of *S*. *aureus* transmission was from humans to pets. *S*. *aureus* was not considered as a true commensal of companion dogs and cats, but as an opportunistic human pathogen, which has only been transferred to them [67, 77]. Still little is known about the colonisation patterns of *S*. *aureus* in pets and thus there is a need for additional longitudinal studies. The relative ease with which *S*. *aureus* bacteria can transfer and adapt to new hosts is an example of when a ‘One Health’ approach has to be applied to inhibit the spread of the organism and its acquisition of further antibiotic resistance. Future studies should be conducted to determine the frequency of colonisation at different anatomical locations in pets, which could help to standardise sampling procedures and control prevalence in pet animals. Finally, there is also a need to develop protocols to prevent the spread of *S*. *aureus* and its transference by bacteria antibiotic resistance in households, as well as in veterinary hospitals.
